# Seizure Outcome and Its Prognostic Predictors After Hemispherotomy in Children With Refractory Epilepsy in a Chinese Pediatric Epileptic Center

**DOI:** 10.3389/fneur.2019.00880

**Published:** 2019-08-14

**Authors:** Taoyun Ji, Ming Liu, Shuang Wang, Qingzhu Liu, Ye Wu, Yuehua Zhang, Xinhua Bao, Wen Wang, Ruofan Wang, Guojing Yu, Xiaoyan Liu, Lixin Cai, Yuwu Jiang

**Affiliations:** ^1^Department of Pediatrics, Peking University First Hospital, Beijing, China; ^2^Department of Pediatric Epilepsy Center, Peking University First Hospital, Beijing, China

**Keywords:** children, refractory epilepsy, hemispherotomy, seizure outcome, predictor

## Abstract

**Object:** To explore the post-hemispherotomy seizure outcome and its prognostic predictors in children with refractory epilepsy.

**Methods:** We reviewed 83 consecutive child patients with refractory epilepsy who underwent a hemispherectomy from June 2014 to January 2017 at our Pediatric Epilepsy Center. Demographic, clinical, EEG, neuroimaging, and surgical data were collected. Seizure outcome data were collected via outpatient clinics as well as telephone visits and were graded according to Engel criteria. Logistic regression model and Cox proportional hazard regression model were, respectively, applied to explore the related factors predicting the seizure outcomes of children after a hemispherotomy.

**Results:** Of the 83 patients, 55 (63.2%) were male. The mean seizure onset age was 1.9 years (0–8.7 years), and the mean surgery age was 5 years (0.8–14 years). At a mean follow-up of 3 years, 69 children (83.1%) were seizure free, and 14 (16.9%) exhibited seizure recurrence. In a univariate analysis, whether or not considering follow-up time, a non-lateralized interictal EEG pattern, bilateral PET abnormalities and acute postoperative seizures (APOS) could all predict poor seizure outcomes post-hemispherotomy. Bilateral PET abnormalities were independently correlated with unfavorable seizure outcomes in the multivariate Logistic regression analysis (Odds ratio(*OR*) = 13.05, 95%*CI* = 1.52–112.29*, P* = 0.019) and in the multivariate Cox proportional hazard analysis(*OR* = 13.99, 95%*CI* = 2.75–71.17, *P* = 0.001).

**Conclusions:** Child epileptic patients with bilateral PET abnormalities may have poor seizure outcomes after a hemispherotomy procedure. This study will facilitate better candidate selection for hemispherotomies and early identification of unfavorable seizure outcomes.

## Introduction

Hemispherectomy is an effective surgical approach for medically intractable epilepsy in children with multilobar or hemispheric epileptogenic lesions, such as encephalomalacia, Rasmussen encephalitis, and malformation of cortical development (1, 2). In published large-sample studies, the rate of seizure remission after hemispherectomy varies from 65 to 80% (3–7).Many researchers attempted to identify prognostic predictors for postoperative seizure outcomes. However, inconsistent predictors have been reported in different studies. Predictors such as the etiology of epilepsy (6), contralateral MRI findings (4, 8), electroencephalography (EEG) findings (7), and surgical techniques (9) have been primarily discussed. However, other potential factors that may affect seizure outcomes including seizure semiology (7) and acute postoperative seizures (APOS) (7) received little attention. The goals of our study were to investigate seizure outcomes in a cohort of Chinese children who underwent peri-insular hemispherotomies, and to explore the potential predictors of the seizure outcomes.

## Methods

### Patients

We retrospectively reviewed the data of a cohort of consecutive children who underwent peri-insular hemispherotomies for medically refractory epilepsy in the Pediatric Epilepsy Center of Peking University First Hospital between June 2014 and January 2017. Eighty-three children who were followed up at least 2 years after surgery were enrolled. This study was approved by the institutional review board of Ethic Committee in Peking University First Hospital and a written informed consent was given by all the participants.

### Etiology

The etiology of epilepsy was classified as three types according to neuroimaging and histopathology results: (1) developmental form (hemimegalencephaly or other malformations of cortical development), (2) acquired form (stable and non-progressive encephalomalacia, such as ischaemic strokes, prior intracerebral hemorrhage, asymmetric hypoxic ischaemic injury, prior head trauma, and so on), and (3) progressive form (Rasmussen encephalitis and Sturge Weber Syndrome).

### Basic Characteristics

Demographics, age of seizure onset, age of surgery, course of epilepsy, seizure frequency, and the number of preoperative antiepileptic medicine were recorded. The developmental status before seizure onset was dichotomous as normal or delayed. Seizure frequency was determined according to parental and EEG reports.

### Preoperative EEG and Semiology

Interictal/ictal scalp EEGs were recorded using a video-EEG(vEEG) monitoring system (Nihon Kohden; Japan); the electrodes were arranged according to the international 10–20 system. The duration of vEEG monitoring ranged from 1 to 7 days, and at least three habitual seizures were recorded for each patient. We attempted to document every seizure type if the patient had more than one type of seizure. Seizure types were classified by two epileptologists according to the vEEG findings.

Interictal and ictal vEEG recordings were categorized as follows: (1) Normal (no interictal abnormalities), (2) Ipsilateral (interictal and ictal EEG abnormalities ipsilateral to the lesion side), (3) Non-lateral (contralateral abnormalities or bilateral abnormalities). Contralateral abnormalities referred to slow background, epileptiform discharges and independent ictal onsets. Bilateral abnormalities referred to interictal discharges of nearly equal frequency over bi-hemisphere non-lateralizing ictal onsets.

### Preoperative Neuroimaging

All patients (*n* = 83) underwent 3T high-resolution brain MRI (Philips Achieva, Dutch) dedicated to seizure focus identification. Subtle contralateral abnormalities such as small single white matter lesions, myelination anomalies or isolated areas of subtle T2 hyperintensity without ventricular dilatation, tissue loss, and cortical/subcortical involvement were not further analyzed. Fifty-one patients underwent an interictal FDG-PET (Philips Gemini GXL, Dutch) scan. MRI and FDG-PET results were categorized as follows: (1) unilateral (multilobar structural or metabolic abnormalities limited to one hemisphere) and (2) bilateral (structural or metabolic abnormalities observed on the contralateral hemisphere).

### Peri-Insular Hemispherotomy Surgery

All resections were performed by the same chief neurosurgeon (Lx Cai) using the peri-insular hemispherotomy technique. The surgical side, postoperative complications, and the presence of APOS were recorded. APOS were defined as seizures within 7 days after surgery confirmed by vEEG.

### Follow-Up

The protocol for postoperative follow-up in our epilepsy center refers to visits at 3 and 6 months followed by yearly visits after the operations, while more frequent follow-up visits are required for patients with recurrent seizures. Postoperative seizure status was assessed using a structured questionnaire and was confirmed against medical records via telephone and outpatient visits. The late remission was defined as seizure-recurrence after surgery, but absence of any seizures for at least more than 2 year at last follow-up. Seizure outcomes were described according to the Engel criteria (10), and the postoperative number of antiepileptic drugs was documented from the last medical records during follow-up.

### Statistical Analysis

Categorical variables were summarized as counts and percentages of the total number of patients in each category. Continuous variables were expressed as the means with standard deviations or medians with ranges. Regardless of follow-up time, the initial comparisons between the seizure-free subgroup with seizure-recurrence subgroup were performed univariably using the Wilcoxon rank sum, χ^2^ or Fisher's exact tests. Variables with a significance level above 10% in the initial univariate analysis were then tested in a multivariate Logistic regression model. In consideration of follow-up time, Kaplan-Meier survival analysis was performed to reveal the probability of seizure-free status in the overall group and to explore the univariably potential predictor of surgical outcome; then, a multivariate Cox proportional hazards regression model was applied with consideration of each significant risk factor. Statistical significance was evaluated using the log-rank test and comparison of the 95% confidence intervals (CIs) in Kaplan-Meier survival analysis. *P*-values <0.05 indicated statistical significance, and *P*-values > 5% but <10% were reported to reflect trends. All statistical analyses were performed using SPSS 20.0 (IBM, USA).

## Results

### Clinical Features

#### Demographics

Our cohort consisted of 55 male and 28 female children. The mean age of seizure onset was 1.9 years (standard deviation [SD] 2.2, range 0.0–8.7), the mean duration of epileptic course prior to surgery was 3.1 years (SD 2.6, range 0.2–13.8), and the mean age of surgery was 5.0 years (SD 3.4, range 0.8–14.0).

#### Etiology

A total of 23 patients (27.7%) had developmental forms of epilepsy, while 44 (53%) and 16 (19.3%) patients had acquired forms and progressive forms of epilepsy, respectively. In developmental etiology subgroup, hemimegalencephaly and polymicrogyria were, respectively, found in 6 of 23 patients (26.1%), focal cortical dysplasia in 21.7% (5/23), pachygyria in 17.4% (4/23), and heterotopias were found in 2 patients. Rasmussen encephalitis were in 12 of 16 patients (75%), Sturge Weber syndrome in 18.8% (3/16), and Parry Romberg syndrome was diagnosed in one patient.

#### Semiology

Eighty-two patients (98.8%) experienced partial seizures and 12 (14.5%) showed generalized seizures which may be not genuine generalized seizures. A total of 48.2% (40/83) of the patients exhibited epileptic spasms. Eighteen (21.7%) patients had current or previous status epilepticus, and 13.3% (11/83) of the patients had epilepsia partialis continua (EPC).

#### Preoperative EEG Findings

Ipsilateral abnormalities confirmed by interictal EEG were identified in 49 patients (59.0%). The remaining 34 (41.0%) patients showed bilateral interictal epileptic discharges or a contralateral anomaly. Four patients (4.8%) demonstrated electrical status epilepticus during sleep (ESES). On ictal EEG 77.1% (64/83) of the patients presented ipsilateral onset and the other 19 (22.9%) patients had an independent contralateral ictal EEG onset or bilateral ictal EEG onset.

#### Neuroimaging Findings

Significant contralateral MRI abnormalities were identified in 19 child participants (22.9%). White matter abnormalities were found in 16 of 19 patients (84.2%), lateral ventriculomegaly in 36.8% (7/19), and encephalomalacia was seen in 31.6% (6/19). Minor abnormal gyrus morphology and cortex signal was, respectively, noted in one patient (5.3%). Preoperative PET-CT were performed in 51 (61.4%) patients, 15.7% (8/51) of whom showed bilateral abnormalities.

#### Peri-Insular Hemispherotomy Surgery

Of 83 procedures, 46 were left-sided and 37 were right-sided. One patient experienced focal cortical resection prior to peri-insular hemispherotomy surgery due to intracerebral hemorrhage. In our cohort, no perioperative mortality was observed and APOS were noted in 7 patients (8.4%).

### Surgical Outcomes

The mean follow-up period was 3.0 years (SD0.8, median 2.9 years). Twenty-one children (25.3%) were followed up for more than 4 years; 28 (33.7%) had a follow-up period between 3 and 4 years; and 34 (40.9%) were followed up for 2 to 3 years. At the last visit of follow-up (*n* = 83), 69 children (83.1%) were seizure-free after surgery (Engel class I) and 14 (16.9%) patients experienced seizure recurrence, including one with late remission, one with Engel class II, four with Engel class III, and five with Engel class IV outcomes. The seizure-free rate was 84.3% (70/83) upon the last follow-up.

**Figure 1:**
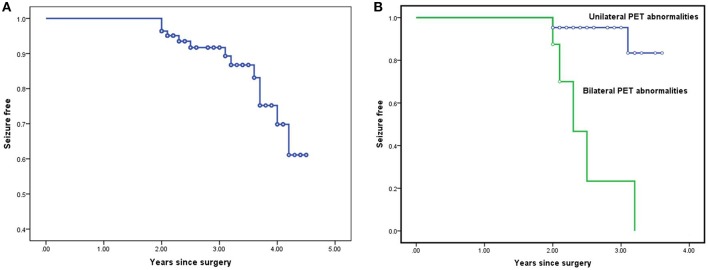
Survival curves for the entire cohort and for the subgroups in Cox proportional hazard regression model: Kaplan-Meier survival curves estimating seizure-free rates at various postoperative time intervals in the entire cohort **(A)**. Kaplan-Meier survival analysis of the potential predictor bilateral PET abnormalities **(B)**.

According to Kaplan-Meier survival curves (Figure 1), the estimated seizure-free rates were 91.8% (±3.3%) at 2 year, 75.3% (±7.4%) at 3 years, and 61.2% (±11.1%) at 4 years after surgery, respectively. However, the actual seizure-free rates were 86.1% (71/83) at 2 years, 82.0% (40/49) at 3 years, and 83.3% (17/21) at 4 years and beyond.

#### Univariate Analysis (Table 1)

To explore the predictors, the patients were divided into 2 groups: seizure-free group and non-seizure-free group. Ignoring the impact of variables such as follow-up duration on the whole model, a non-lateralized interictal EEG pattern (*P* = 0.006), bilateral PET abnormalities (*P* = 0.001), and APOS (*P* = 0.01) were statistically correlated with seizure recurrence after surgery (*P*-values all <0.05) in univariate analysis.

**Table 1:** Univariate analysis of potential predictors on seizure outcome.

	**Overall (*n* = 83)**	**Seizure-free (*n* = 70)**	**Not Seizure-free (*n* = 13)**	*P-Value	***P*-Value of Log-Rank test in Kaplan Meier analysis**
**Gender**					
Male	55 (66.3)	45	10	0.528	0.356
Female	28 (33.7)	25	3		
Mean age at seizure onset, y	1.9 (±2.2)	1.9 (±2.3)	1.6 (±1.7)	0.678	–
Mean age at surgery, y	5.0 (±3.4)	5.0 (±3.3)	5.3 (±4.0)	0.747	–
Mean seizure duration, y	3.1 (±2.6)	3.0 (±2.5)	3.7 (±3.3)	0.399	–
Positive perinatal history	6 (7.2)	5 (7.1)	1 (7.7)	1.000	0.892
Positive family history	5 (6.0)	5 (7.1)	0	1.000	0.241
**Development before seizure onset, n (%)**					
Normal	40 (48.2)	33 (47.1)	7 (53.8)	0.766	0.619
Delay	43 (51.8)	37 (52.9)	6 (46.1)		
**Antiepileptic drugs (SD)**					
Pre-operative	2.7 (±1.1)	2.7 (±1.1)	2.8 (±0.7)	0.587	–
Post-operative	1.6 (±0.9)	1.5 (±0.7)	2.3 (±1.3)	0.001	–
**Seizure frequency**					
<1 seizure per day	2 (2.4)	1 (1.4)	1 (7.7)	0.287	0.986
1–5 seizures per day	32 (38.6)	29 (41.4)	3 (23.1)		
>5 seizures per day	49 (59.0)	40 (57.2)	9 (69.2)		
Etiology					
Developmental	23 (27.7)	20/23 (86.9)	3/23 (13.1)	0.096	0.218
Acquired	44 (53.0)	34/44 (77.3)	10/44 (22.7)		
Progressive	16 (19.3)	16/16(100)	0/16 (0)		
**Seizure type or epileptic syndrome, n (%)**					
Simple partial seizure	82 (98.8)	69 (98.6)	13 (100)	1.000	0.768
Complex partial seizure	17 (20.5)	16 (22.9)	1 (7.7)	0.286	0.161
Generalized seizure	12 (14.5)	9 (12.9)	3 (23.1)	0.280	0.845
Spasms	40 (48.2)	33 (47.1)	7 (53.8)	0.766	0.220
Status epilepticus	18 (21.7)	16 (22.9)	2 (15.4)	0.724	0.424
ESES/CSWS	4 (4.8)	4 (5.7)	0	1.000	0.325
EPC	11 (13.3)	11 (15.7)	0	0.199	0.126
**Interictal EEG, n (%)**					
Ipsilateral	49 (59.0)	46 (65.7)	3 (23.1)	0.006	0.038
Non-lateralized	34 (41.0)	24 (34.3)	10 (76.9)		
**Ictal EEG, n (%)**					
Ipsilateral onset	64 (77.1)	57 (81.4)	7 (53.8)	0.065	0.357
Non-lateralized onset	19 (22.9)	13 (18.6)	6 (46.2)		
**Cerebral MRI, n (%)**					
Ipsilateral abnormal	64 (77.1)	57 (81.4)	7 (53.8)	0.065	0.389
Bilateral abnormal	19 (22.9)	13 (18.6)	6 (46.2)		
**PET-CT(*****n*** **=** **51), n(%)**		*n* = 43	*n* = 8	0.001	<0.001
Ipsilateral abnormal	43 (84.3)	40 (93.0)	3 (37.5)		
Bilateral abnormal	8 (15.7)	3 (7.0)	5 (62.5)		
**Hemispherectomy, n (%)**					
Left side	46 (55.4)	38 (54.3)	8 (61.5)	0.765	0.529
Right side	37 (44.6)	32 (45.7)	5 (38.5)		
**APOS, n (%)**					
Absent	76 (91.6)	67 (95.7)	9 (69.2)	0.010	0.003
Present	7 (8.4)	3 (4.3)	4 (30.8)		

* *P-value was calculated by using the Wilcoxon rank sum, χ^2^, and Fisher's exact tests to compare seizure-free group with seizure-recurrence group in univariate analysis, ignore the effect of variable of follow-up time. APOS, acute postoperative seizures*.

Taking follow-up duration into consideration, a non-lateralized interictal EEG pattern (*P* = 0.038), bilateral PET abnormalities (*P* < 0.001), and APOS (*P* = 0.003) were also correlated with postoperative seizure recurrence using Log-Rank test in Kaplan Meier analysis.

#### Multivariate Analysis

Applying multivariate Logistic regression analysis (Supplemental Table 1), bilateral PET abnormalities were independently correlated with non-seizure-free status (*P* = 0.019, odds ratio(*OR*) = 13.05, 95%*CI* = 1.52–112.29).

By means of multivariate Cox proportional hazard analysis (Supplemental Table 2), bilateral PET abnormalities tended to independently predict poor prognosis of surgery (*P* = 0.001), and the patients with bilateral PET abnormalities were nearly 14 times more likely to undergo a failed peri-insular hemispherotomy (OR = 13.99, 95%*CI* = 2.75–71.17, *P* = 0.001).

## Discussion

In this study, we investigated the postoperative outcomes of 83 eligible patients with refractory epilepsy, hemisphere resection and postoperative follow-ups of at least 2 years. Among them, the seizure-free rates were 86.1% at 2 years, 82.0% at 3 years and 83.3% at 4 years and beyond. The mean duration of the follow-up was 3.0 years (2–4.5 years), with 83.1% (69/83) of the patients achieving seizure-free status. The seizure-free rates were similar to previously reported rates ranging from 63 to 80% in 5 large series (*n* = 84–170) (3–7). One longitudinal seizure outcome study showed that long-term outcomes change minimally over 2 to 11 years after surgery (3), which may be attributable to the selection criteria and the definition of a good prognosis for epilepsy surgery. Our study found that the seizure-free rate following hemispherical surgery decreased rapidly within 2 years (−3.1%) and decreased slightly over 2 years (−2.7%). However, the short follow-up duration was a limitation of this study and should be extended in further studies.

In this study, both of univariate and multivariate analyses methods were applied to explore the predictive factors of seizure outcomes. In the univariate analysis, a non-ipsilateralized interictal EEG pattern, bilateral PET abnormalities, and APOS appeared to potentially predict seizure recurrence after surgery (*P* < 0.05). In the multivariate analysis, bilateral PET abnormalities were the only predictor independently correlated with the unfavorable outcome of epilepsy surgery (*P-*values was 0.019 or 0.001).

### Interictal EEG Pattern

One meta-analysis suggested that lateralized findings on interictal (*OR* = 1.66, 95%*CI* = 1.03–2.67, *P* = 0.04) or ictal (*OR* = 1.88, 95% *CI* = 1.15–3.07, *P* = 0.01) EEG may correlate with favorable seizure outcomes (11). The results of this study coincided with those of some previous studies. However, some other studies showed no correlation between interictal or ictal EEG and seizure outcomes (7). As far as seizure semiology was concerned (3, 7), the presence of generalized seizures was related to unfavorable seizure outcomes (*OR* = 1.84, 95% *CI* = 1.18–2.89, *P* = 0.008). It is reported that the immature structural and functional brains of younger children are more likely to experience generalized seizures than adult brains (12, 13). However, we did not determine any correlation between the presence of generalized seizures and unfavorable prognosis, although younger children constituted a large proportion of the cases in this study.

### APOS

Acute seizures within 1 week after surgery were considered as an indication of postoperative epilepsy recurrence. In a large series of 170 children who underwent peri-insular hemispherotomy, 19% (31/163) of the children experienced APOS, and 71% (22/31) of them had poor postoperative seizure outcomes (7). Another study on seizure outcomes after extratemporal resections and hemispherectomy suggested consistent results that APOS could predict a poor long-term seizure outcome (14). In our study, 8.4% (7/83) of the patients had APOS, and a similar correlation between APOS and seizure recurrence was determined in univariate analysis (*P* = 0.027). But in the multivariate analysis, APOS could not independently predict seizure outcomes.

### Bilateral PET Abnormalities

Only one study with a large sample size of hemispherectomy patients focused on bilateral PET abnormalities (7). The study revealed that bilateral PET abnormalities were associated with unfavorable outcomes (OR = 2.53, 95%CI = 1.02–5.85) and that they may be more reliable markers of potential independent epileptogenicity than bilateral structural abnormalities on brain MRI (7). In another study of 8 children with hemimegalencephaly, bilateral PET abnormalities were also proved to be a predictor for unfavorable seizure outcomes (15). In contrast, in a small sample of 18 infants who underwent hemispherectomy, no correlations were identified between poor seizure outcomes and bilateral PET abnormalities (16). In the present study, 8 out of the 51 children patients (15.7%) who underwent preoperative PET-CT scan exhibited bilateral PET abnormalities and 5 of them (62.5%, 5/8) had unfavorable seizure outcomes. It is suggested that patients with preoperative bilateral PET abnormalities should be carefully evaluated before hemispherotomy surgery and closely followed up after the operation.

Besides the potential predictors mentioned above, other related factors such as contralateral MRI anomalies (8), etiology (6) and epileptic surgical procedure (10) were reported to be probably associated with poor seizure outcome.

### The Contralateral Anomaly of Cerebral MRI

The value of preoperative contralateral MRI anomalies to predict the seizure outcomes after hemispherectomy therapy for epileptic patients were explored by a number of studies with inconsistent results. A study enrolling 110 patients who underwent hemispherectomy displayed no obvious correlation between contralateral MRI anomalies and postoperative seizure recurrence (8); while another research involving 43 patients reviewing hemispherectomy therapy suggested that contralateral MRI anomalies could predict the postoperative epilepsy recurrence (4). In our study, no correlation was identified between bilateral MRI abnormalities and postoperative epilepsy outcomes. Contralateral MRI abnormalities were foci of the preoperative evaluations before hemispherectomy. The structural abnormalities displayed in brain MRI, such as cortical malformations, abnormal white matter signals, abnormal white matter myelination and encephalomalacia, were supposed to be associated with poor seizure outcomes; but further investigations were still needed because of the limited number of relevant studies. In our opinion, contralateral MRI abnormalities were not an absolute contraindication for hemispherectomy therapy. Surgical candidacy should be determined by utilizing a combinative evaluation protocol including semiology analysis, preoperative vEEG, PET-CT as well as neuropsychological assessment. Emergent or early-stage MRI was helpful for determining the location and extent of lesions at early stage in favor of explaining clinical manifestations, especially when the patients had acquired etiology such as encephalitis and hemorrhage.

### Etiology

One earlier research study found that patients with malformations of cortical development may have less favorable outcomes (6), but this has not been observed in other studies (3, 5, 7). In our study, different results were found in seizure outcomes of etiology. Progressive etiology had the best prognosis (100%, 16/16), followed by developmental etiology (90%, 18/20), and acquired etiology (80%, 32/40) was the worst. Possible explanations include different criteria for patient selection and various surgical techniques in different epilepsy centers. In a Cleveland epilepsy center, approximately 11.2% (19/170) of patients had undergone prior focal cortical resection (3). The integrity of excision or hemispherical disconnection also affects the surgical prognosis.

### Epilepsy Surgery

In a previous study (10), the surgical technique was believed to be unrelated to the postoperative seizure outcomes (*OR* = 1.51, 95% *CI* 0.96–2.36, *P* = 0.07). As mentioned above, all children in our study underwent peri-insular hemispherotomy by the same chief surgeon, and only one patient had received prior focal cortical resection. The surgeries underwent by the same senior epileptic surgical specialist could eliminate the individual variations of surgical technique; and the works of a single epileptic center during a short period could reduce deviations produced by the different evaluative conception and the update of medical equipment. No perioperative or postoperative mortality was observed. However, we did not review or analyze surgical techniques, prior surgeries or other complications.

## Conclusion

Hemispherotomy was an effective and safe surgical option to relieve the seizure burden of candidates with intractable epilepsy. The seizure-free rates after hemispherotomy were approximately 84.3% at a mean follow-up of 3 years. This study will facilitate better candidate selection for hemispherotomy and early identification of unfavorable seizure outcomes.

## Data Availability

All datasets generated for this study are included in the manuscript/Supplementary Files.

## Author Contributions

YJ and LC designed the study and revised the paper. TJ and ML drafted the paper, analyzed and interpreted the data, and submitted the paper. WW, RW, and GY analyzed and interpreted the data. YJ, XL, LC, YW, YZ, XB, QL, SW, and TJ collected the patients.

### Conflict of Interest Statement

The authors declare that the research was conducted in the absence of any commercial or financial relationships that could be construed as a potential conflict of interest.
