# Supplementary material for: Seizure Outcome and Its Prognostic Predictors After Hemispherotomy in Children With Refractory Epilepsy in a Chinese Pediatric Epileptic Center
Source: Front Neurol. 2019 Aug 14;10:880. doi: 10.3389/fneur.2019.00880 (PMC6702354; doi:10.3389/fneur.2019.00880)
Supplement: Supplementary file 1 [file Table_1.DOCX]

**Supplemental Table 1**: multivariate Logistic regression model of seizure outcome (whole-model *χ^2^*=16.29, *P*=0.023).

| **Variables** | **Risk ratio** | **95.0% Confidence Interval** | ***P* value** |
| --- | --- | --- | --- |
| **Non-lateralized interictal EEG** | 2.56 | 0.26-25.61 | 0.43 |
| **Non-lateralized ictal EEG** | 2.49 | 0.23-26.72 | 0.45 |
| **Bilateral MRI abnormalities** | 2.60 | 0.193-34.99 | 0.47 |
| **Bilateral PET abnormalities** | 13.05 | 1.52-112.29 | 0.02 |
| **Acute postoperative seizure** | 2.16 | 0.05-95.62 | 0.69 |
