# Supplementary material for: Seizure Outcome and Its Prognostic Predictors After Hemispherotomy in Children With Refractory Epilepsy in a Chinese Pediatric Epileptic Center
Source: Front Neurol. 2019 Aug 14;10:880. doi: 10.3389/fneur.2019.00880 (PMC6702354; doi:10.3389/fneur.2019.00880)
Supplement: Supplementary file 2 [file Table_2.DOCX]

**Supplemental Table 2**: multivariate Cox proportionalhazard model of seizure outcome (whole-model *χ^2^*=17.98, *P*<0.001).

| **Variables** | **Risk ratio** | **95.0% Confidence Interval** | ***P* value** |
| --- | --- | --- | --- |
| **nonlateralized interictal EEG** | 2.08 | 0.42-10.25 | 0.37 |
| **Bilateral PET abnormalities** | 13.99 | 2.75-71.17 | 0.00 |
| **Acute postoperative seizure** | 0.34 | 0.04-3.23 | 0.35 |
